# Optimization of Cadmium Adsorption onto a Cellulose Acetate–Clay Composite Membrane Using Box–Behnken Design

**DOI:** 10.3390/molecules31172976

**Published:** 2026-08-25

**Authors:** Sihem Dhieb, Safa Gamoudi, Farida Baraka, Xabier Erdocia, Jalel Labidi, Ridha Ben Salem, Younes Moussaoui

**Affiliations:** 1 Organic Chemistry Laboratory (LR17ES08), Faculty of Sciences of Sfax, University of Sfax, Sfax 3029, Tunisia; sihemdhieb98@gmail.com (S.D.);; 2 Faculty of Sciences of Gafsa, University of Gafsa, Gafsa 2112, Tunisia; 3 Laboratory of Composite Materials and Clay Minerals, National Center for Research in Materials Science, Technopole Borj Cedria, B.P. 73, Soliman 8027, Tunisia; safa.gammoudi@eniga.ugaf.tn; 4 Monastir Preparatory Engineering Institute, University of Monastir, Monastir 5019, Tunisia; 5 Chemical and Environmental Engineering Department, Faculty of Engineering of Gipuzkoa, University of the Basque Country UPV/EHU, Plaza Europa 1, 20018 San Sebastian, Spain; 6 Department of Applied Mathematics, Faculty of Engineering of Gipuzkoa, University of the Basque Country UPV/EHU, Plaza Europa 1, 20018 San Sebastian, Spain

**Keywords:** cadmium, membrane, adsorption, box-behnken design, optimization

## Abstract

Cadmium contamination in water poses a serious environmental and health concern due to its high toxicity and persistence. In this context, the development of efficient and low-cost adsorbent materials has attracted increasing attention. This work examines the removal of Cd(II) from aqueous solution using a cellulose acetate–clay composite membrane as an adsorbent material. To evaluate the impact of clay inclusion, membranes were fabricated with varying clay concentrations (0%, 12.5%, and 25%). A Box–Behnken design was used to optimize the process; thermogravimetric analysis, X-ray diffraction, and Fourier-transform infrared spectroscopy were used to analyze the produced composite membranes. The characterization results confirmed the successful incorporation of clay into the cellulose acetate matrix and revealed important changes in the membrane structure and surface morphology. The adsorption performance was strongly affected by operating conditions, particularly temperature, contact time, and clay content. Under the optimal conditions of 30 °C, 4 h, and 5% clay content, the CA-Clay composite membrane achieved a maximum Cd(II) removal efficiency of 93.93% and an adsorption capacity of 12.35 mg/g. These results demonstrate that the composite membrane has a high affinity toward Cd(II) ions, exhibiting its high potential as a low-cost and efficient adsorbent for the removal of Cd(II) from aqueous solution.

## 1. Introduction

Cadmium is a hazardous and persistent heavy metal that can accumulate in the environment and pose significant risks to human health [1,2]. Due its toxicity, the US Environmental Protection Agency and the World Health Organization set a maximum permissible concentration of Cd in drinking water at 0.005 mg/L and 0.003 mg/L respectively [3,4]. This heavy metal occurs naturally and is further released by industrial and mining activities [5,6,7,8,9]. Chronic exposure to Cadmium can affect numerous organs, especially the heart, bones, the kidneys, and lungs [10,11,12,13]. Therefore, effective approaches for Cd(II) is essential.

One of the challenges that humanity faces in ensuring sustainable growth and environmental safety is protecting the environment and human health [14]. For the removal of metal ions, several remediation techniques have been developed with varying degrees of effectiveness. These techniques include chemical precipitation [15], advanced oxidation [16], membrane filtering [17,18,19], adsorption [20,21], solvent extraction [22,23], electrochemical treatment [24,25], coagulation, and flocculation [26,27,28]. Adsorption stands out among these techniques as an efficient physicochemical technique due to its simplicity in handling, low capital cost, high efficiency, and suitability for both batch and continuous processes [29]. Adsorption is a surface phenomenon in which the surface of a solid adsorbent accepts dissolved metal ions from aqueous solution [30]. This phenomenon involves a variety of mechanisms, such as electrostatic attraction, ion exchange, surface complexation, and precipitation [31]. Several operating parameters significantly influence adsorption performance such as pH, temperature and contact time, which control metal speciation in the solution and the availability of active binding sites [32]. A wide range of materials can be used, including biochar [33,34], fly ash [35], orange peel [36], activated carbon [37,38,39], clay [40,41,42], cellulose and its derivatives [43].

Membrane-based adsorption is a potentially effective method for removing heavy metals because it incorporates the advantages of membrane separation and adsorption into a single material [44]. Among the polymeric materials used for membrane fabrication, cellulose acetate is very attractive due to the convenience of processing into porous, hydrophilic membranes and can be functionalized or mixed to adjust its performance [45]. Moreover, its excellent film-forming capacity, biocompatibility, beneficial water permeability and metal ion rejection qualities [46]. Clay and clay composite materials have been developed as extremely effective adsorbents for heavy metals removal from aqueous solution [47]. In particular, the large specific surface area of clay minerals provides numerous active sites for the adsorption of cationic and anionic species [48]. Moreover, their abundance, low cost, negative surface charge, and high cation-exchange capacity make clay minerals attractive fillers for the fabrication of composite membranes and also for Cd(II) adsorption [49]. Consequently, a cellulose acetate-clay composite membrane provides an appropriate and durable approach to removing Cd(II) from aqueous solutions [50].

Finding the optimum operating parameters is crucial to achieving the maximum adsorption efficiency, as the adsorption process is influenced by complex and independent variables [51]. Therefore, process optimization becomes crucial in order to determine the values of design parameters at which the response reaches its optimal level [52]. In this context, statistical optimization tools are required to successfully analyze and explain these complicated connections. Response surface method (RSM) is a combination of statistical and mathematical techniques used to model and analyze various processes in order to determine correlations between experimental inputs and to show the main and interaction effects [53]. Among the various experimental designs used in RSM, the Box–Behnken design (BBD) design is often employed given its effectiveness in reducing the number of experimental runs while allowing accurate estimation of quadratic models and interaction effects [54].

In this work, we present the creation of a cellulose acetate–clay composite membrane and a methodical assessment of its ability to absorb cadmium from aqueous solutions. In order to create a useful mathematical model that was optimized to determine the optimal adsorption conditions, response surface methodology based on Box–Behnken design was used to assess the effects of individual components and their interactions.

## 2. Results and Discussion

### 2.1. Clay Characterization

**Figure 1:**
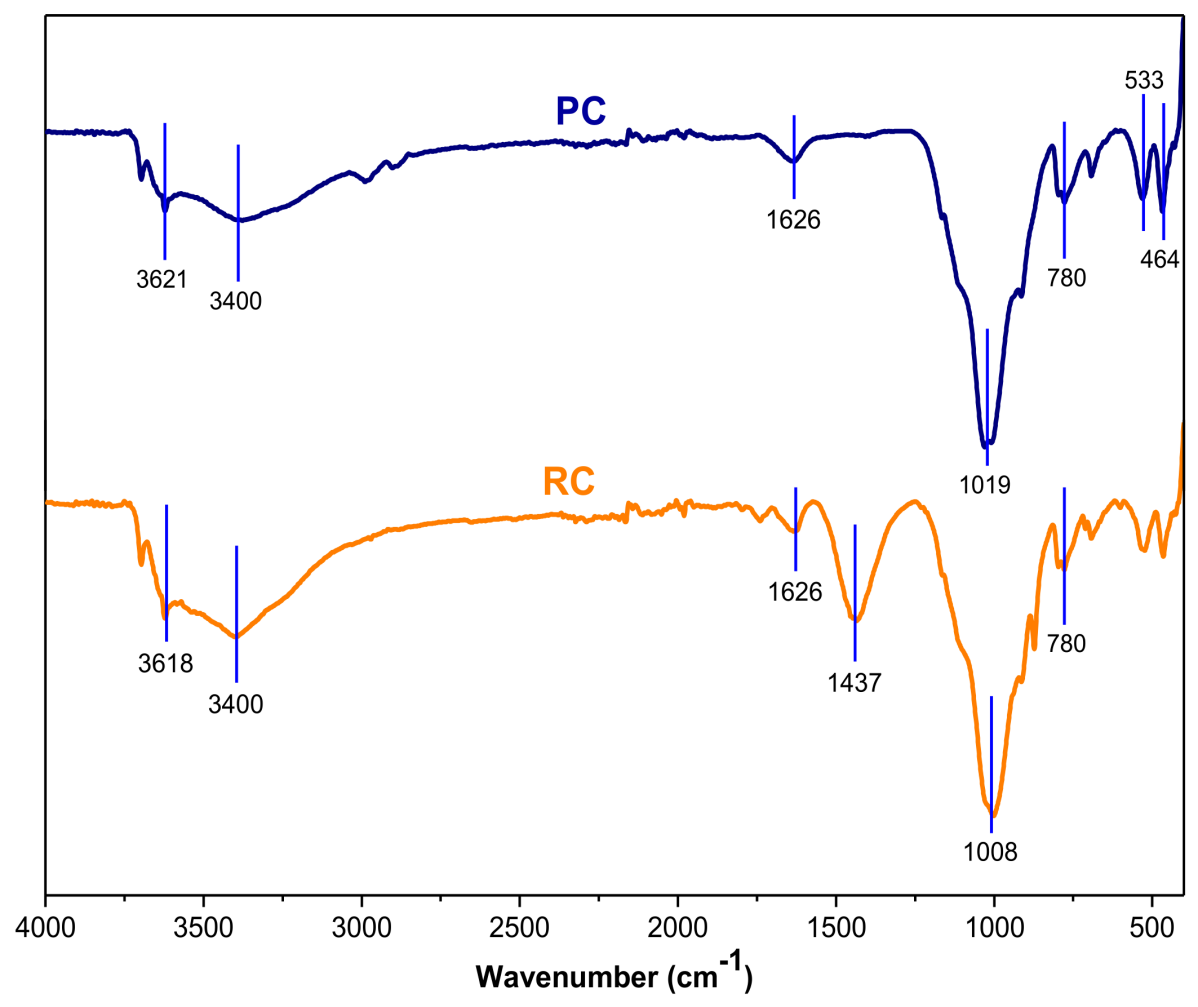
FTIR spectra of raw clay (RC) and purified clay (PC).

For raw clay (RC), the infrared spectra (Figure 1) reveal stretching vibrations at 3618 cm^−1^ for structural OH groups found in the clay samples [55]. The OH stretches of water are classified to the band around 3400 cm^−1^ [56]. The distortion of H_2_O is responsible for the band observed at 1626 cm^−1^ [57]. The carbonate-specific band at about 1437 cm^−1^ is regarded as an impurity [58]. An intense band at 1008 cm^−1^ is attributed to the silicate (Si-O-Si) structure, while other bands at 780, 533, and 464 cm^−1^ may be attributed to octahedral (Al-O-Si), (Al-Al-O), and (Si-O-Si) bending vibrations, respectively [59]. The band at 1431 cm^−1^ vanished in the purified clay (PC) spectrum demonstrates that the purifying procedure was effective [60].

**Figure 2:**
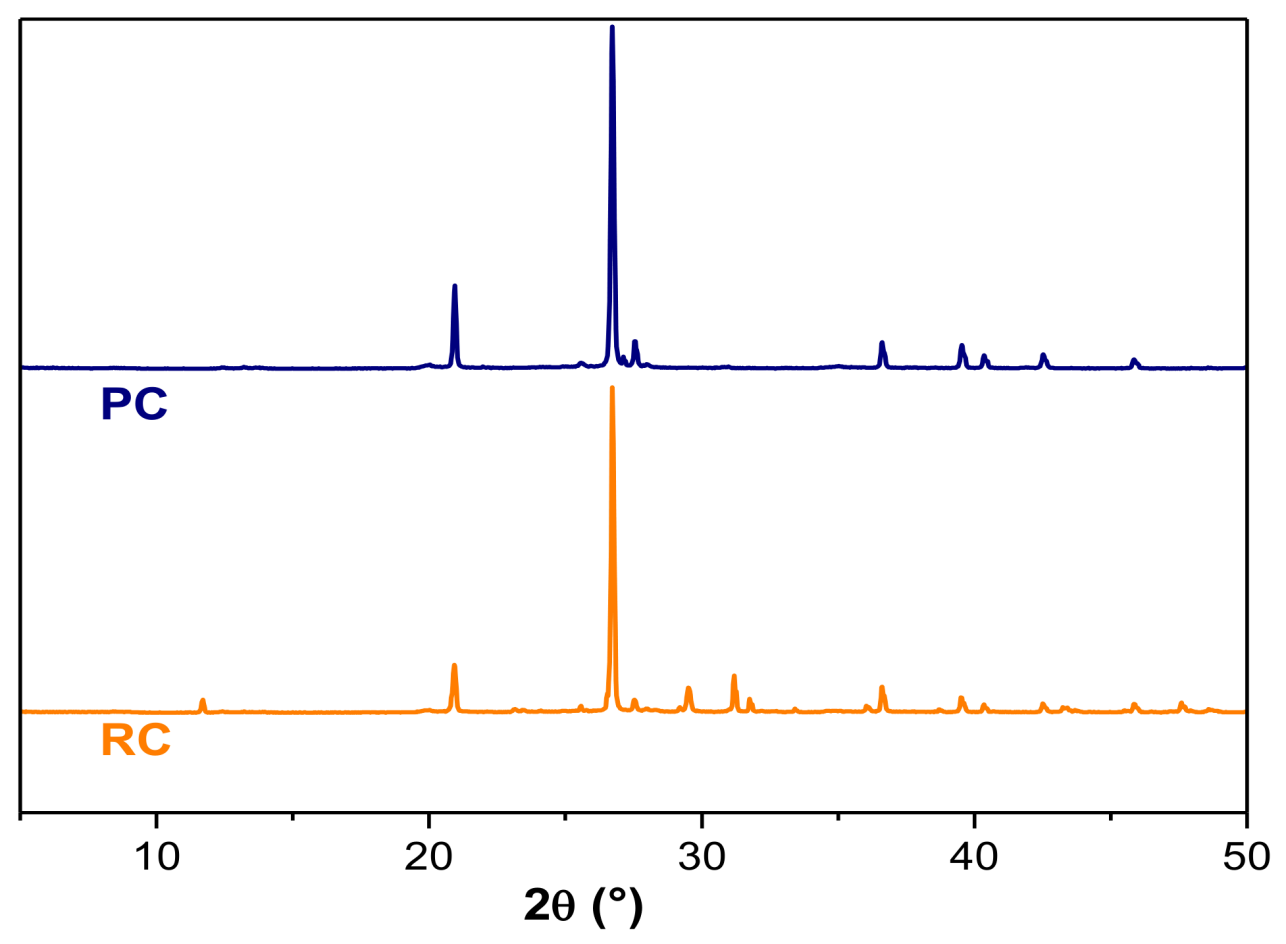
XRD patterns of raw clay (RC), purified clay (PC).

The XRD patterns of raw and purified clay (Figure 2) show a dominant sharp reflection at 26.71° assigned to α-quartz [61], a basal feature at 21° consistent with phyllosilicate (illite/sericite/smectite) family reflections [62], and additional peaks at 36, 39, 40, and 45° attributable to feldspathic minerals [63].

### 2.2. Membrane Characterization Before and After Adsorption

**Figure 3:**
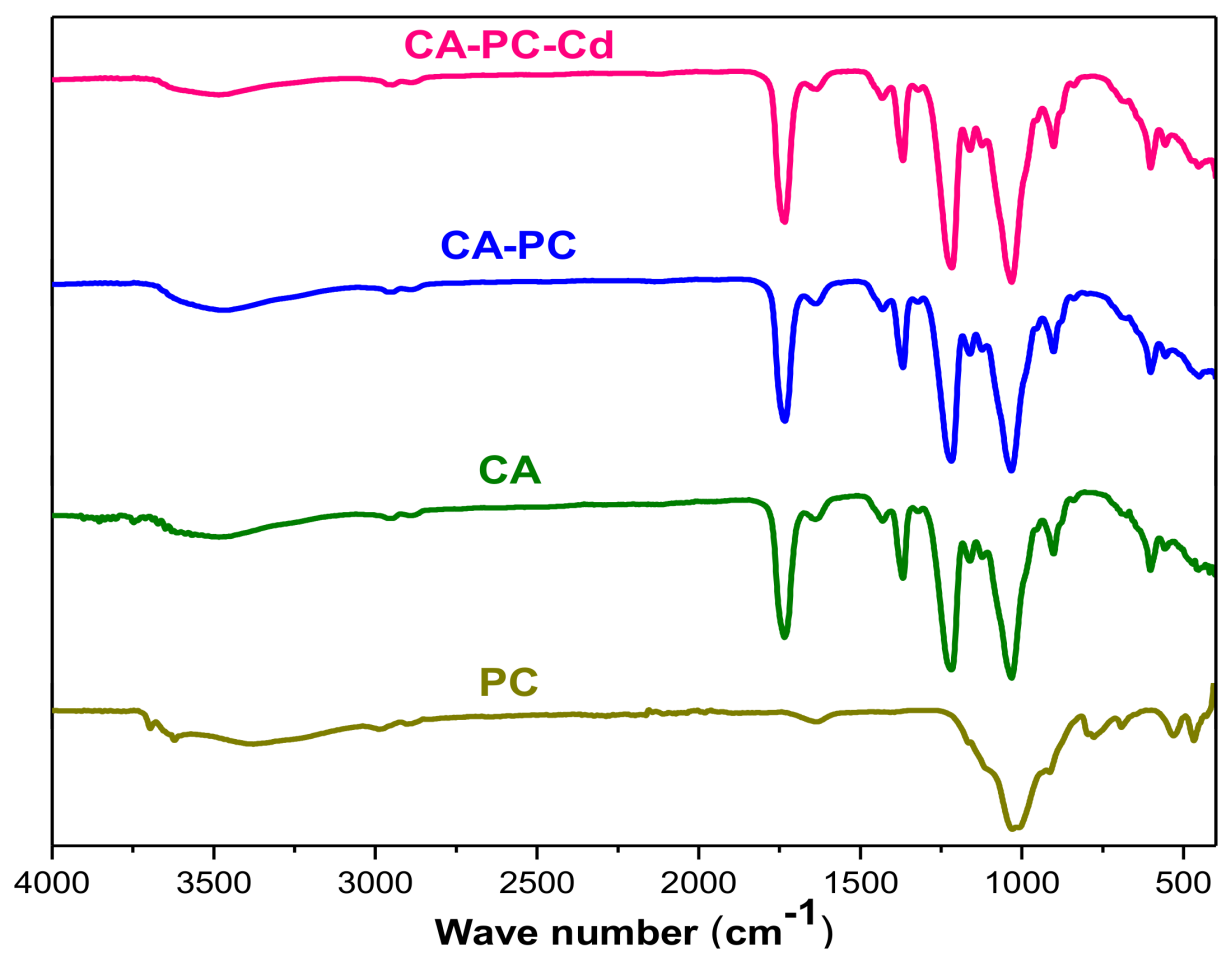
FTIR spectra of membrane before and after adsorption, PC (purified clay); CA (cellulose acetate); CA-PC (membrane cellulose acetate purify clay: before cadmium adsorption); and CA-PC-Cd (membrane cellulose acetate purify clay: after cadmium adsorption).

The FTIR spectra of purify Clay (PC), cellulose acetate (CA), the CA-PC membrane before adsorption and the CA-PC-Cd membrane after Cd(II) adsorption are presented in Figure 3.

The CA spectra exhibit the typical cellulose acetate bands: the ester carbonyl stretching vibration at 1735 cm^−1^, the C-O-C stretching band at 1240 cm^−1^ and the C-O stretching band at 1036 cm^−1^ [64,65,66]. The PC spectrum shows characteristic bands of clay such as the O-H band around 3624 cm^−1^, the Si-O-Si stretching vibration at 1040 cm^−1^ and other bands at 915,519 and 466 cm^−1^ corresponding to Al-O-Si, Al-Al-O and Si-O-Si bending vibrations [67]. In the CA-PC membrane, the presence of the main bands of both components indicates the successful incorporation of the clay into the cellulose acetate matrix. The slight shifts observed may indicate interactions between cellulose acetate functional groups and clay surface hydroxyl groups [67,68,69]. After adsorption of Cd(II) ions, the FTIR spectra (Figure 3) showed no significant changes, suggesting that the adsorption process did not induce major chemical modifications of the membrane functional groups [70]. Moreover, the presence of hydroxyl and carbonyl groups may contribute to Cd(II) adsorption through surface interactions [71]. The weak band observed near 796 cm^−1^ is mainly attributed to quartz-related O-Si-O vibrations. Its appearance or slight variation after Cd(II) adsorption may be associated with the involvement of the clay phase in the adsorption [72,73].

**Figure 4:**
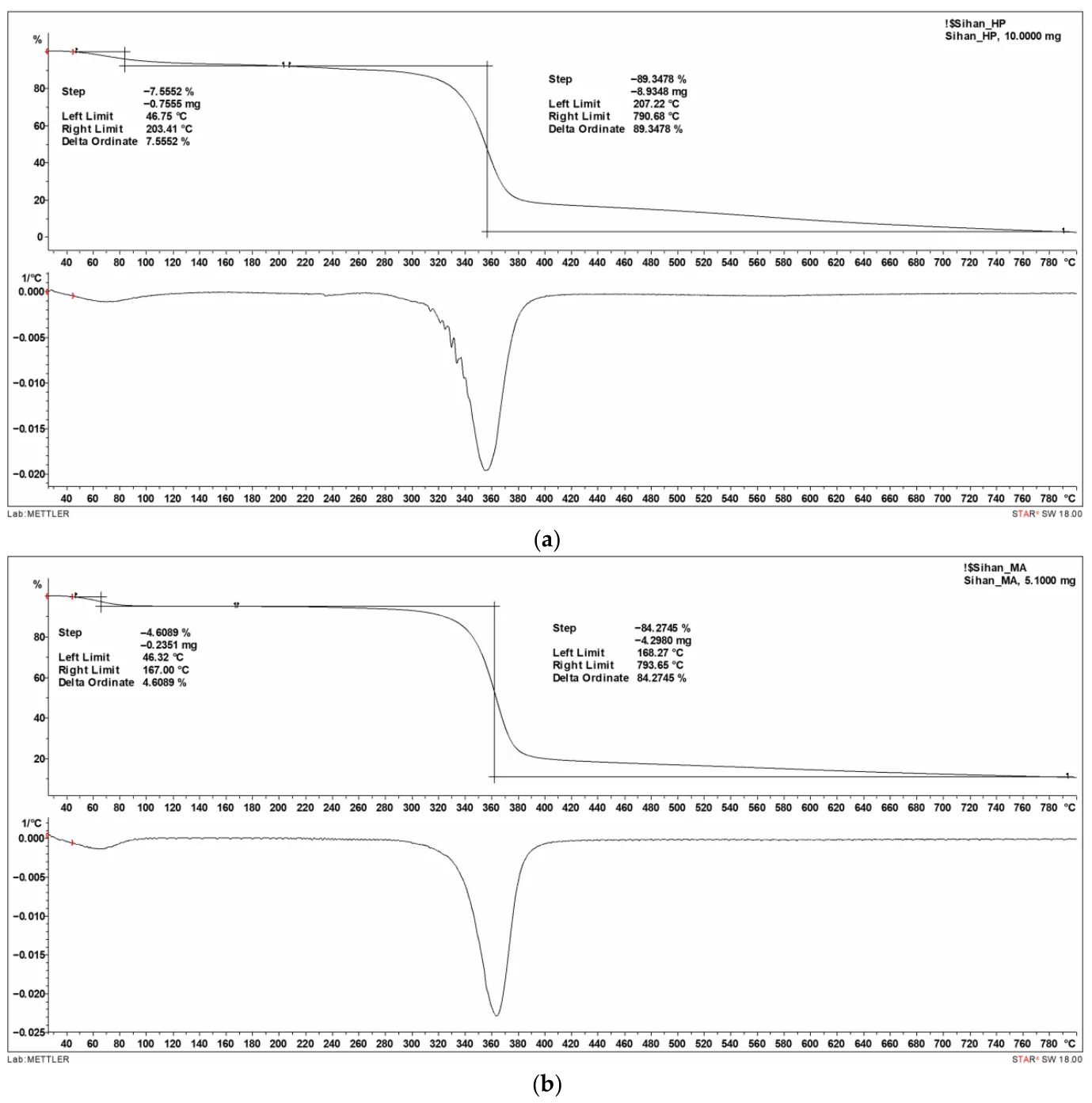
TGA/DTG spectra of membrane (**a**) before and (**b**) after cadmium adsorption.

Thermal analysis of the cellulose-acetate/clay membrane before and after Cd(II) adsorption shows two reproducible regions (Figure 4).

A low-temperature endothermic mass loss below ≈150 °C, assigned to desorption of physiosorbed and bound water, is about 4.08% (≈0.41 mg for a 10.00 mg sample) (Figure 4a) and decreases slightly to ≈3.60% (≈0.36 mg) after Cd uptake (Figure 4b) which may be associated with modifications in the water retention performance after Cd(II) adsorption. The HP sample has an onset near ~267 °C and a DTG peak decomposition around ~310 °C. After adsorption the decomposition region is modified: the onset shifts by ≈+13 °C (onset ≈280 °C) indicating which suggests that Cd(II) may interact with polymer/clay functional sites and alters local thermal stability or decomposition kinetics. Such assignments follow standard interpretations of TGA/DTG for polymeric and composite adsorbents: water loss at low temperature, polymer/clay decomposition, and shifts in peak due to metal–polymer interactions [74]. Thus, thermal analysis corroborates the adsorption results by indicating the involvement of active functional sites in Cd(II) binding [75].

### 2.3. Experimental Design Results

The Box–Behnken experimental design generated a set of 15 runs, and the corresponding adsorption capacities are presented in Table 1. The results were analyzed using a quadratic regression model, and the statistical significance of the model was evaluated by ANOVA employing the Statgraphics Centurion version XVI (Statpoint Technolgies Inc., Warrenton, VA, USA) (Table 2).

The F-value of the model was found to be 2.458, corresponding to a *p*-value of 0.167. Since the *p*-value is higher than 0.05, the model is not statistically significant at the 95% confidence level. This indicates that the variation explained by the model is not sufficiently greater than the residual error. However, the coefficient of determination (R^2^) was 0.8157, indicating that 81.6% of the variability in Cd(II) adsorption capacities can be explained by the model. This relatively high R^2^ value suggests a good agreement between experimental and predicted values, although it should be interpreted with caution given the lack of statistical significance of the model. This discrepancy may be attributed to the variability inherent to the adsorption system. The relationship between the independent variables and Cd(II) adsorption capacity was described by the following second-order polynomial equation (in coded variables):

(1) $$y=11.29-0.035X_{1}-0.005X_{2}+0.305X_{3}+0.140X_{1}X_{2}-0.232X_{1}X_{3}-0.270X_{2}X_{3}-0.217X_{1}^{2}-0.308X_{2}^{2}-0.346X_{3}^{2}$$

The magnitude and sign of the coefficients provide insight into the effect of each variable. Among the linear terms, contact time (X_3_) exhibits the strongest positive effect on Cd(II) adsorption capacity, while temperature (X_1_) and clay content (X_2_) show relatively minor negative contributions. The negative coefficients of the quadratic terms indicate the presence of curvature in the response surface, confirming that optimal adsorption occurs at intermediate levels of the variables rather than at their extreme values. Interaction terms also play a relevant role, particularly the negative interactions between temperature–time (X_1_X_3_) and clay content–time (X_2_X_3_), suggesting that the simultaneous increase in these variables may reduce adsorption efficiency beyond certain conditions.

**Figure 5:**
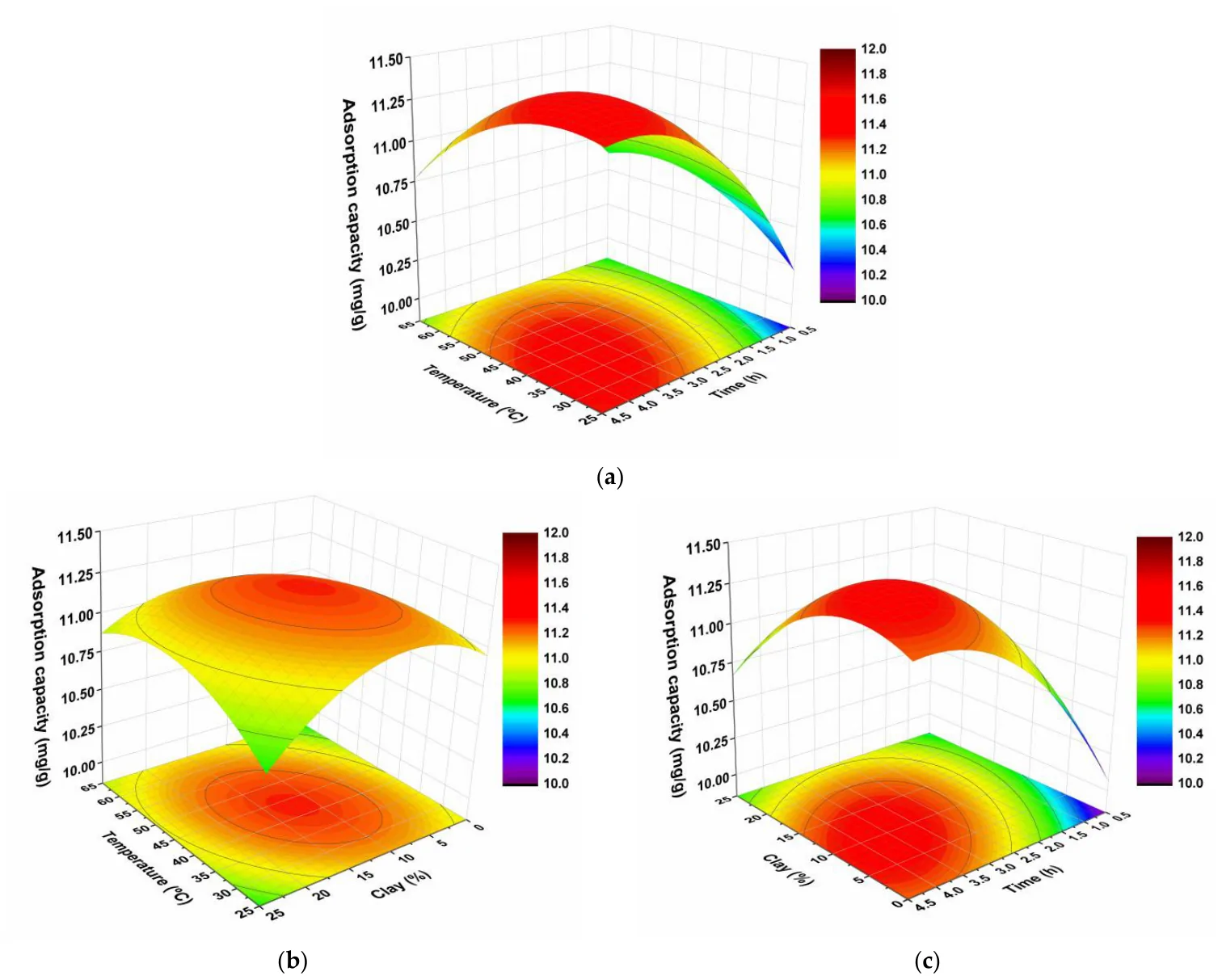
Three-dimensional response surface plots illustrating the interaction effects of process variables on Cd(II) adsorption capacity: (**a**) clay content and temperature, (**b**) temperature and contact time, and (**c**) clay content and contact time.

Despite the limitations of the model, the response surface analysis provides valuable insight into the combined effects of the selected variables. The three-dimensional response surface plots (Figure 5) highlight the interactions between temperature, clay content, and contact time.

As shown in Figure 5a, the adsorption capacity rises from 10.6 mg/g at low clay contents (0–5%) and high temperatures (60–65 °C) to values above 11 mg/g under intermediate conditions. This behaviour indicates that both temperature and clay content positively influence Cd(II) adsorption capacity up to an optimum range. This increase can be related to the adsorption sites provided by the clay phase and enhanced Cd(II) diffusion at moderate temperatures. However, further increases in clay content do not improve adsorption performance and may even lead to a slight decrease, possible due to particle aggregation and reduced accessibility of active sites [76].

In Figure 5b, the combined effect of temperature and contact time is shown. The adsorption capacity increases from approximately 10.3 mg/g at short contact time (<1 h) and a low temperature (25–30 °C) to nearly 11.2 mg/g during longer contact times (3–4 h) and temperatures around 35–40 °C. The increase with contact time may be related to the progressive diffusion of Cd (II) ions from the solution into the membrane accompanied by a slower uptake as adsorption equilibrium approaches [70,73]. The modest decrease in adsorption at higher temperatures may show a temperature-dependent modification to the adsorption equilibrium.

Similarly, Figure 5c shows that adsorption capacity increases with contact time and medium-low content (5–15%), reaching values close to 11.2 mg/g at moderate clay contents (5–15%) and a moderate contact time (3–4 h). The increase could be the result of both progressive Cd(II) mass transfer toward these sites and new adsorption sites provided by the clay phase. Reduced accessibility of adsorption sites at higher clay loading and progressive occupation of the accessible sites may be the cause of the limited improvement outside the optimal region [73,76]. This suggests that optimal adsorption occurs at intermediate conditions, where a balance between adsorption site availability and mass transfer is achieved.

Overall, these results indicate that the Cd(II) adsorption capacity is governed by the combined effect and non-linear effects of temperature, contact time, and clay content, rather than by the influence of a single parameter alone. These observations are consistent with the regression model, in which the negative coefficients of the quadratic terms indicate the existence of an optimal region within the experimental domain.

The response surface patterns are consistent with previous studies on clay-based adsorbents for the removal of Cd(II). Previous studies indicate that Cd(II) adsorption increases initially with contact time and then slows down as equilibrium is approached, while increasing clay content often enhances adsorption until the available sites become occupied [77,78]. The intermediate optimum found by the Box–Behnken design is in accordance with these patterns; however, the optimum conditions may change based on the characteristics of the adsorbent and the experimental conditions.

To determine the optimal operating conditions for maximum Cd(II) adsorption capacity, the developed quadratic model was used to perform numerical optimization based on response surface methodology. It should be noted that, because the quadratic model was not statistically significant, the predicted optimum should be considered tentative rather than definitive. Therefore, the experimental validation performed under these conditions provides preliminary evidence of improved Cd(II) adsorption capacity. The optimal operating conditions for Cd(II) adsorption capacity using the cellulose acetate predicted by the model are compiled in Table 3.

To validate the model, experiments were conducted under the predicted optimal conditions, yielding an experimental removal efficiency of 12.35 mg/g (shown in Table 3). The higher experimental value compared to the predicted one suggests that the model correctly identifies the optimal region, although some deviation is observed, likely due to experimental variability or limitations of the model.

### 2.4. Comparative Study

Table 4 compares the Cd(II) adsorption capacity of CA-Clay composite membrane with those of several adsorbents that have been documented in the literature. The different types of adsorbents have variable reported adsorption capacity. These differences might be explained by variations in the chemical characteristics of the adsorbents as well as the nature and accessibility of adsorption sites [79]. Furthermore, differences in experimental parameters, such as pH, temperature, initial concentration, contact time, and adsorbent dosage may influence the measured adsorption capacity [30]. The CA–clay composite membrane exhibited a higher Cd(II) adsorption capacity than several adsorbents reported in the literature. This relatively high adsorption capacity may be explained by the combined effect of the clay phase and the cellulose acetate matrix, which may provide complementary adsorption sites, enhancing cadmium ion migration and adsorption [80,81].

### 2.5. Desorption

**Figure 6:**
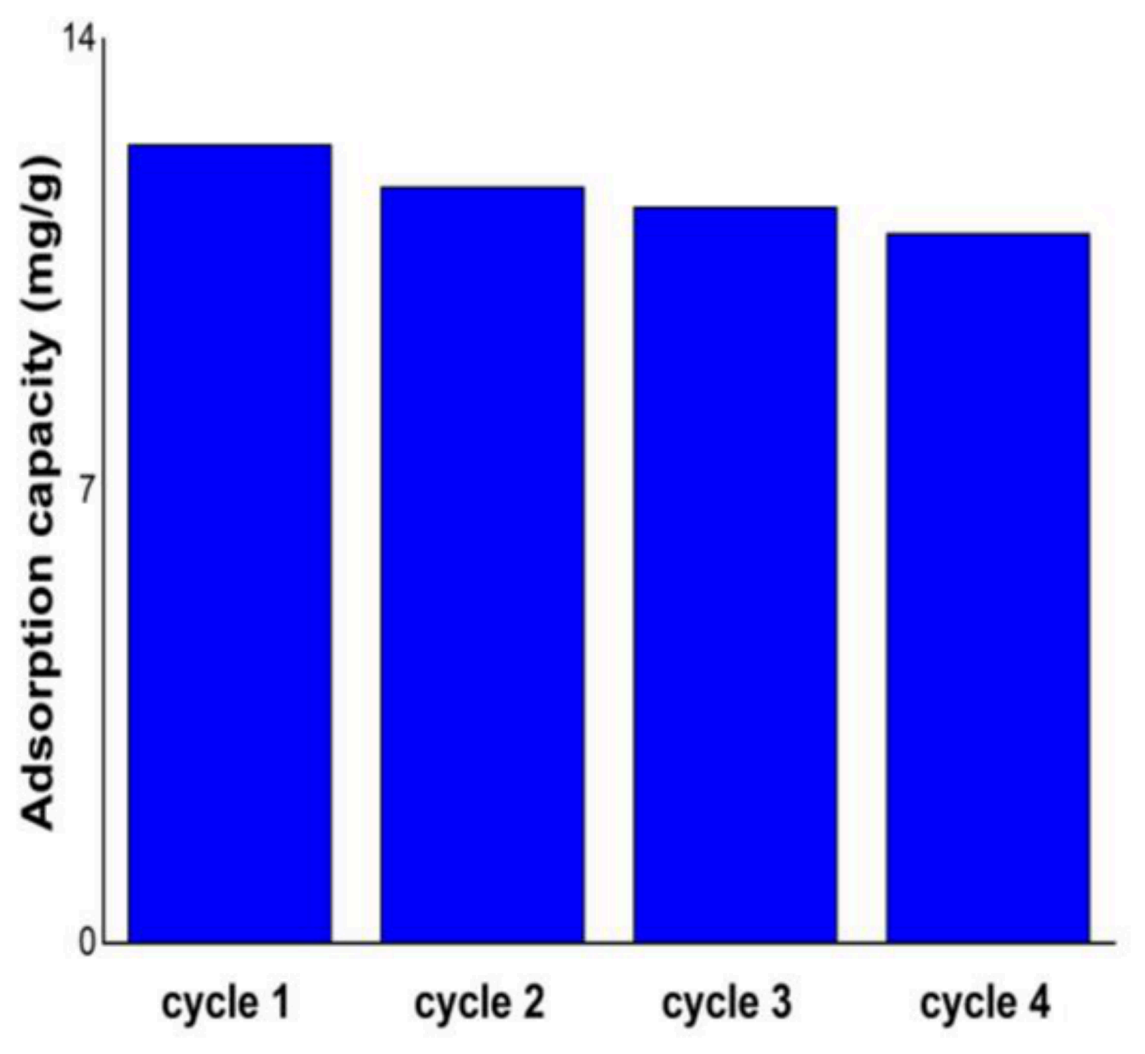
Adsorption and desorption cycles.

The regeneration of the membrane was carried out using the acidic method [88], and the results are shown in Figure 6.

In fact, the membrane already used for cadmium adsorption is placed in 50 mL of HCl (0.05 mol L^−1^) and stirred at 300 rpm. Before being dried, the sample was cleaned three times with distilled water to get rid of hydrochloric acid or cadmium ions after each adsorption or desorption procedure [89]. As shown in Figure 6 the Cd(II) removal efficiency decreased from 12.35 mg/g in the first cycle to 11.70 mg/g, 11.39 mg/g and 10.98 mg/g after four cycles. This gradual decrease in adsorption capacity may be attributed to the partial loss of active adsorption sites, incomplete desorption of Cd(II) ions, or slight structural alterations of the membrane after repeated regeneration cycles. Nevertheless, the relatively high adsorption capacity maintained after four cycles demonstrates the good reusability and stability of the membrane, highlighting its potential.

## 3. Materials and Methods

### 3.1. Materials and Reagents

The chemicals used in this study included sulfuric acid (H_2_SO_4_, 96%), acetic acid (CH_3_COOH 99.8%), and hydrochloric acid (HCl, 37%), Cadmium nitrate tetrahydrate (Cd(NO_3_)_2_·4H_2_O, 98%), Cellulose acetate), and Dithizone (diphenylthiocarbazone),were purchased from Sigma-Aldrich (Darmstadt, Germany) without additional purification.

The clay minerals used in this study was collected from Maknassi, Sidi Bouzid, Tunisia.

### 3.2. Clay Purification

10 g of clay was mixed with 100 mL of HCl (2 M). The mixture was stirred at 550 rpm for 3 h at 25 °C. After treatment, the suspension was filtered and the residue was dried in an oven at 100 °C for 24 h [90].

### 3.3. Preparation of Membrane

Composite membranes were prepared by incorporating clay at different loadings: 0%, 12.5%, and 25%. For each formulation, the necessary amount of clay was dissolved in 2 mL of acetic acid and shaken for an hour at 500 rpm. To guarantee uniform dispersion, the mixture was ultrasonically agitated for 10 min. The mixture was then agitated for three hours after the addition of cellulose acetate and 3 mL of acetic acid. The resultant homogeneous solution was subsequently poured into a Petri dish and kept in an incubator set at 40 °C and 40 rpm over 8 h.

### 3.4. Characterization Methods

The mineralogical composition and structural modifications of the raw and purified clay samples were examined using X-ray diffraction (XRD) analysis. A Phillips X’Pert PRO diffractometer (Phillips N.V., Amsterdam, The Netherlands) running at 40 kV and 40 mA with Cu-Kα radiation (λ = 1.5418 Å) was used to record the diffraction patterns.

The thermogravimetric analysis (TGA) was carried out using a Mettler Toledo instrument (Mettler Toledo, Columbus, OH, USA). Membrane samples with masses between 5 and 10 mg (before and after Cd(II) adsorption) were heated in an atmosphere of nitrogen (50 mL min^−1^) from 30 to 800 °C at a rate of 10 °C min^−1^.

A Perkin Elmer spectrum Tow FTIR spectrometer (Perkin Elmer, Shelton, CT, USA) fitted with a diamond crystal and a Universal Attenuated Total Reflectance (ATR) accessory was used to record the Fourier Transform Infrared (FTIR) spectra of the raw clay, purified clay and membrane samples both before and after Cd(II) adsorption. With a resolution of 8 cm^−1^, spectra were gathered between 400 and 4000 cm^−1^.

### 3.5. Batch Adsorption Studies

Adsorption tests were performed using 50 mL of a cadmium aqueous solution with an initial concentration of 50 mg/L at pH = 6. Membrane was placed in the solution and stirred at 300 rpm for indicated contact time from 0.5 to 4.5 h.

After the adsorption experiments, each membrane was carefully collected and placed in a sterile Petri dish for further characterization. The residual cadmium concentration in solution was determined using the dithizone complexation method. Shortly, 10 mL of Cd(II)solution (50 mg/L) was acidified to pH = 2 by adding 1 mL of H_2_SO_4_ (0.5 M). Subsequently, the dithizone solution (1 mg/L) was added at a molar ratio of 2:1 (dithizone:Cd(II)). The formed Cd–dithizone complex was quantified by measuring its absorbance [91].The adsorption percentage and adsorption capacity were calculated using the following equations:

(2) $$R(\%)=\frac{C_{0}-C_{e}}{C_{0}}\times 100$$

$$q=\frac{\left(C_{0}-C_{e}\right)V}{M}$$

where C_0_ and C_e_ are respectively the initial and equilibrium concentration, V is the volume of metal solution, and M is the mass of the adsorbent.

### 3.6. Box–Behnken Design

One popular design for response surface methodology that is very helpful for determining cause-and-effect linkages between inputs and responses in experiments is the Box–Behnken design (BBD) [92]. To maximize the efficiency of heavy metal removal and preparation conditions, a factorial experimental design was employed. Table 5 shows the three factors used: clay percentage, contact time, and temperature.

The model used is a Box–Behnken design. The number of experience N is 15 experiences calculated using the flowing equation:

(3) $$N=2k*\left(k-1\right)+C_{0}$$

where k is a number of factors and C_0_ is the number of central points [93].

The second-order polynomial, such as the Box–Behnken design, can be approximated using the following equation:

(4) $$y=\;\beta_{0}+\sum_{i=1}^{3}\beta_{i}x_{i}+\sum_{i=1}^{3}\beta_{ii}x_{i}^{2}+\sum \sum_{i<j=2}^{3}\beta_{ij}x_{i}x_{j}+\varepsilon$$

where y denotes the response variable (adsorption capacity), x_i_ corresponds to the levels of the independent variables, *β*_i_ represents the linear contribution of the i-th factor, *β*_ii_ accounts for its quadratic effect, *β*_ij_ describes the interaction between the i-th and j-th factors, and ε is the random error term [94].

## 4. Conclusions

This study successfully developed and assessed a cellulose acetate/clay composite membrane for cadmium ions removal from aqueous solutions. The successful integration of clay into the polymer matrix was verified by the structural characterization using FTIR, which also showed the presence of functional groups that may contribute to Cd(II) adsorption. TGA/DTG studies showed that the composite membrane has good thermal stability with discernible changes following cadmium adsorption, suggesting changes in the thermal behaviour of the membrane after cadmium adsorption. The adsorption process was investigated using a Box–Behnken design, which allowed the identification of a tentative optimal region. The response surface analysis showed that Cd (II) adsorption capacity was influenced by temperature, contact time, and clay content. Although the model was not statistically significant at the 95% confidence level, it provided useful information for identifying the optimal operating region. A maximum cadmium adsorption capacity of 12.35 mg/g corresponding to a removal efficiency of 93.93% was attained under optimal conditions (T = 30 °C; Contact time = 4 h and clay percentage = 5%), demonstrating the composite membrane’s excellent efficacy. The process of Cd(II) adsorption is probably controlled by a combination of surface interactions such as electrostatic attraction and ion exchange and the functional groups present in the cellulose acetate–clay composite membrane.

Overall, the results show that the cellulose acetate/clay composite membrane is a promising and effective material for removing cadmium from aquatic environments. To further confirm its practical potential, future research may concentrate on regeneration and actual wastewater applications.

## Figures and Tables

**Table 1:** Box–Behnken design matrix with three independent variables and experimental Cd(II)adsorption capacities.

Run	Temperature (X_1_)	Clay-Percentage (X_2_)	Time (X_3_)	Temperature (°C)	Clay Percentage (%)	Time (h)	Adsorption Capacity (mg/g)
1	0	0	0	45	12.5	2.5	11.19
2	0	1	−1	45	25	0.5	10.37
3	1	0	−1	65	12.5	0.5	10.72
4	0	0	0	45	12.5	2.5	11.34
5	−1	1	0	25	25	2.5	10.98
6	1	1	0	65	25	2.5	10.98
7	−1	0	1	25	12.5	4.5	11.19
8	1	−1	0	65	0	2.5	10.27
9	−1	0	−1	25	12.5	0.5	10.12
10	0	0	0	45	12.5	2.5	10.32
11	0	−1	−1	45	0	0.5	10.29
12	1	0	1	65	12.5	4.5	10.87
13	0	1	1	45	25	4.5	10.44
14	−1	−1	0	25	0	2.5	10.83
15	0	−1	1	45	0	4.5	11.43

**Table 2:** Analysis of variance (ANOVA) for the quadratic regression model.

Sources	Degree of Freedom	Sum of Squares	Mean Square	F	*p*-Value
Regression	9	2.1817	0.2424	2.458	0.167
Residuals	5	0.4929	0.0986	-	-
Total	14	2.6746	-	-	-

**Table 3:** Optimum conditions for maximizing the Cd(II) adsorption capacity.

		Adsorption Capacity (mg/g)	
Variables	Optimum Values	Predicted	Experimental
Temperature	30 °C	11.44	12.35
Contact time	4 h		
Clay percentage	5%		

**Table 4:** Comparative Cd(II) removal performance of various adsorbents.

Adsorbent	Adsorption Capacity (mg/g)	References
Porous resins	3.33	[82]
Clays	4	[83]
Natural Clays	5.65	[73]
Natural clays	5.85	[70]
Biochars derived from agricultural	6.28	[84]
*Auraucaria heteropylla*	9.25	[85]
Agriwaste-Biosorbent	10	[86]
Silk/Betonite Clay Composite	11.35	[87]
Composite cellulose acetate clay	12.35	This work

**Table 5:** Experimental variables and level settings.

Variable	Coding		Levels	
		−1	0	+1
Temperature (°C)	X_1_	25	45	65
Clay percentage (%)	X_2_	0	12.5	25
Contact time (h)	X_3_	0.5	2.5	4.5

## Data Availability

The original contributions presented in this study are included in the article. Further inquiries can be directed to the corresponding author.
